# Attempts to grow human noroviruses, a sapovirus, and a bovine norovirus in vitro

**DOI:** 10.1371/journal.pone.0178157

**Published:** 2018-02-13

**Authors:** Tomoichiro Oka, Garrett T. Stoltzfus, Chelsea Zhu, Kwonil Jung, Qiuhong Wang, Linda J. Saif

**Affiliations:** 1 Food Animal Health Research Program, Ohio Agricultural Research and Development Center, Department of Veterinary Preventive Medicine, The Ohio State University, Wooster, OH, United States of America; 2 Department of Virology II, National Institute of Infectious Diseases, Tokyo, Japan; University of Michigan, UNITED STATES

## Abstract

Noroviruses (NoVs) and Sapoviruses (SaVs) are enteric caliciviruses that have been detected in multiple mammalian species, including humans. Currently, efficient cell culture systems have been established only for murine NoVs and porcine SaV Cowden strain. Establishment of an efficient *in vitro* cell culture system for other NoVs and SaVs remains challenging; however, human NoV (HuNoV) replication in 3D cultured Caco-2 cells and a clone of Caco-2 cells, C2BBe1, human enteroids and in human B cells has been reported. In this study, we tested various cells and culture conditions to grow HuNoVs and a human SaV (HuSaV) to test the possibility of the propagation in different cells and culture conditions. We also attempted to grow a bovine NoV (BoNoV) in *ex vivo* organ cultures. We did not observe significant RNA level increases for HuSaV and BoNoV under our test conditions. HuNoV RNA levels increased to a maximum of ~600-fold in long-term Caco-2 cells that were cultured for 1–2 months in multi-well plates and inoculated with HuNoV-positive and bacteria-free human stool suspensions using serum-free medium supplemented with the bile acid, GCDCA. However, this positive result was inconsistent. Our results demonstrated that HuNoVs, BoNoV and HuSaV largely failed to grow *in vitro* under our test conditions. Our purpose is to share our findings with other researchers with the goal to develop efficient, reproducible simplified and cost-effective culture systems for human and animal NoVs and SaVs in the future.

## Introduction

Noroviruses (NoVs) and Sapoviruses (SaVs) are non-enveloped, single stranded, positive sense RNA viruses of the family *Caliciviridae*. Based on the complete capsid sequences, NoVs and SaVs are classified into at least five genogroups (GI, GII, GIII, GIV, and GV) [1] and 15 genogroups (GI-GXV), respectively [2], both of which are further divided into multiple genotypes [3, 4]. NoVs have been detected from humans, swine, cattle, sheep, rodents, cats, dogs, and lions [5]. SaVs have been detected from humans, pigs, mink, dogs, sea lions, bats, chimpanzees, rodents, and carnivores [2, 6].

Human NoVs (HuNoVs) have been recognized for over four decades. A single genotype, genogroup II and genotype 4 (GII.4), is the leading cause of acute non-bacterial gastroenteritis in humans worldwide since around 2000 [7]. Although human volunteer studies showed that histopathological changes occurred in the small intestine of HuNoV-infected individuals [8], the cell type(s) in which human HuNoVs replicate in immunocompetent individuals are still unknown. Recently, HuNoVs were reported to replicate in the intestinal enterocytes of immunocompromised transplant patients [9]. Both HuNoV structural protein VP1 and non-structural (RNA polymerase and genome-linked viral protein VPg) antigens were detected in the intestinal epithelial cells, providing direct evidence of viral replication in these cells. Although HuNoV antigens were also detected in immune cells in the lamina propia, proof of definitive HuNoV replication in these immune cells was lacking because an epithelial cell marker was also detected simultaneously in those cells, suggesting that these immune cells contained HuNoV antigens acquired via phagocytosis of HuNoV-infected epithelial cells.

Efficient in vitro cell culture systems have been achieved for murine NoVs using murine macrophage cell lines (e.g., RAW264.7), primary macrophages and dendritic cells [10], and mouse B cell lines [11]. Murine NoV RNA titers in the RAW264.7 cell supernatant increased > 6 log_10_ within 4 days post-inoculation [12]. HuNoV RNA levels in feces in acute gastroenteritis were extremely high (up to 12 log_10_ genomic RNA copies/g or mL of stool) [13], despite their low infectious dose levels (approximately 1~3 × 10^3^ genomic copies) [14]. However, the reported HuNoV RNA increases in 3D cultures of differentiating Caco-2 or a derivative cell line CBBe2 ranged from 2–3 log_10_ based on qRT-PCR [15, 16]. During our study and manuscript preparation, two new methods using human B cells or human intestinal enteroids have been reported for successful propagation of HuNoVs *in vitro*, but also with 2–3 log_10_ increased RNA levels based on qRT-PCR [11, 17, 18].

Human SaVs (HuSaVs) also have been recognized for over four decades and cause acute non-bacterial gastroenteritis in humans; however, their infection site(s) and the cell type(s) that are susceptible to HuSaVs *in vivo* remain unknown [4]. A single genotype, genogroup I and genotype 2 (GI.2), was predominantly detected from acute non-bacterial gastroenteritis outbreaks throughout Japan in 2012 and 2013 [19]. The reported propagation of HuSaV in African green monkey kidney cells and primary human embryo kidney cells has been noted but not confirmed [20, 21]. Currently, an efficient cell culture system has been established only for porcine origin SaVs (GIII strains) using the porcine kidney cell lines, LLC-PK1, and bile acids in the culture medium [22–25].

In this study, we attempted to propagate HuNoVs, a HuSaV, and a bovine NoV (BoNoV) in multiple cell types and using various culture conditions. Although most of these trials failed, we detected increased HuNoV RNA levels once during our study when a sterile mixture of HuNoV GII.4 positive stool specimens was inoculated onto long-term cultured monolayers of Caco-2 cells.

## Materials and methods

### Fecal specimens

The following HuNoV-positive stool samples: GI.1/Norwalk [GenBank Accession Number M87661], GII.2/HS255 [KJ407074], GII.4/HS66 (US95-96 cluster) [KJ407076], GII.4/HS194 (Den_Haag_2006b cluster) [GU325839], GII.4/HS288 (New_Orleans_2009 cluster) [KJ407075], and GII.4/HS292 (New_Orleans_2009 cluster) [KJ407073], and GII.6/HS245 [KJ407072]) were diluted as 10% (w/v) suspension in sterile MEM and vortexed vigorously, then centrifuged at 1,800 × g for 30 min, and sterilized through 0.22 μm-pore size filters.

Three other HuNoV GII.4 positive stool specimens: two strains and one strain in New_Orleans_2009 cluster and Den_Haag_2006b cluster, respectively, and a HuSaV GI.2-positive stool specimen was diluted as 10% (w/v) suspension in sterile MEM and vortexed vigorously, and then mixed with 1/10 volume of chloroform and shaked for 20 min using a mechanical shaker. The mixture was further centrifuged at 1,500 x g for 20 min. The supernatant was collected as sterilized stool suspension. This treatment protocol was routinely used for the preparation of stool suspension for enterovirus isolation in cultured cells at the Department of Virology II, National Institute of Infectious Diseases.

Capsid sequence-based HuNoV genotyping and GII.4 cluster assignment for the above HuNoVs were performed using the online NoV genotyping tool of NoroNet (http://www.rivm.nl/mpf/norovirus/typingtool/) [3, 26]. Capsid sequence-based HuSaV genotyping was performed based on phylogenetic analysis using reference sequences and the method described previously [4, 27].

Twenty seven HuNoV-positive stool samples [GII.1, n = 1; GII.2, n = 1; GII.3, n = 2; GII.4, n = 4 (2 New Orleans cluster and 2 Sydney cluster); GII.5, n = 1; GII.6, n = 2; GII.7, n = 2; GII.8, n = 1; GII.9, n = 1; GII.12, n = 2; GII.13, n = 2; GII.14, n = 2; GII.15, n = 2; GII.16, n = 2; and GII.17, n = 2] were provided by Dr. Jan Vinje at Division of Viral Diseases, Centers for Disease Control and Prevention, Atlanta, GA, USA. They were diluted as 10% (w/v) suspension in sterile MEM and vortexed vigorously, then centrifuged at 2,000 × g for 30 min, and the supernatant was sterilized through 0.22 μm-pore size filters.

The large intestinal contents of a Gnotobiotic (Gn) calf inoculated with Bo/GIII.2/CV186-OH/00/US strain (GenBank accession no. AF542084) [28, 29] was stored at -80°C until use. The sample was diluted 10-fold in Dulbecco’s phosphate-buffered saline without Mg^2+^ and Ca^2+^ [PBS (-), Sigma, St. Louis, MO], vortexed briefly, and centrifuged at 4,000 × g at 4°C for 30 min. The supernatants were further centrifuged at 10,000 × g at 4°C for 3 min and filtered through 0.2 μm-pore size filters.

All of these sterilized HuNoV-, HuSaV-, or BoNoV-positive fecal suspensions were aliquoted to individual tubes, stored at -70°C and thawed only once for cell culture trials.

### RNA extraction

The RNA of fecal suspensions and cell culture samples (cell supernatant or supernatant of the mixture of cell supernatant and cell lysates) were extracted using RNeasy Mini kit (QIAGEN, Valencia, CA, USA) or MagMAX-96 Viral 1 Kit (Ambion, Austin, TX) in combination with the RNA extraction robot MagMax^TM^ Express (Applied Biosystems, Foster City, CA), respectively, according to the manufacturer's instructions.

### TaqMan RT-qPCR or RT-PCR

The viral RNA titer of human or bovine NoVs in the fecal and cell culture samples was determined using real-time reverse transcription-PCR (RT-qPCR) assays. Human GI and GII NoVs were analysed by RT-qPCR with COG1F and COG1R primers and RING1a and RING1b probes (FAM-), and COG2F and COG2R primers and RING2 probe (FAM-), respectively [30], using one-step RT-PCR kit (Qiagen) and MasterCycler RealPlex2 (Eppendorf) and the following conditions: 50°C for 30 min for reverse transcription, then 95°C for 15 min followed by 45 cycles of a two-step PCR (95°C for 15 sec and 56°C for 60 sec) as described [31]. Bovine GIII NoV was quantified with primers SWGIIInewF and SWGIIIrev and SWGIII probe (FAM-) [28] at the following reaction conditions: 50°C for 30 min. then 95°C for 15 min followed by 45 cycles of a 2-step PCR: 95°C for 15 sec, 60°C for 60 sec.

Human SaV was quantified with primers SaV124F, 1F, 5F, and 1245R and probes (FAM-) SaV 124TP and SaV 5TP [32] under the following conditions: 50°C for 30 min for reverse transcription, and then 95°C for 15 min followed by 40 cycles of a 2-step PCR: 95°C for 15 sec, 56°C for 60 sec.

### Cell lines, primary cells and tissues

For HuNoV and HuSaV culture trials, we used four human cell cultures: 1) Human primary intestinal epithelial cells (HIEC) (a gift from Dr. Jean-Francois Beaulieu, Université de Sherbrooke, Québec, Canada) [33]; 2) human colonic epithelial cell line HT29-Cl.16E [a gift from Dr. Christian L. Laboisse Professor, Nantes University School of Medicine, France, and provided through Dr. Ulrich Hopfer (Case Western Reserve University, Cleveland, OH, USA)] [34]; 3) human colorectal adenocarcinoma cell line Caco-2 cells (ATCC #HTB-37); and 4) B-cell derived cell line, JVM-2 cells (ATCC CRL-3002). We also used a porcine small intestinal (jejunum) epithelial cell line, IPEC-J2 (a gift from Dr. Bruce D. Schultz Lab, Kansas State Univ, USA) [35], a monkey kidney cell line, Vero BI (a gift from Dr. Louis Harris, Boehringer Ingelheim Co., Ltd., Ingelheim, Germany), for HuNoV culture trials.

For BoNoV culture trials, the organ segments from duodenum, jejunum and ileum were collected aseptically from a neonatal Gn calf. Primary cells from the ileal mucosa were prepared by Thermolysin digestion method as described [33] and checked cell viabilities (cell shape, light reflection, etc.) microscopically. Calf intestinal mononuclear cells [MNCs] from ileum and peripheral blood mononuclear cells [PBMC]) were prepared as described previously [36].

### Cell culture conditions

HIEC cells were grown in OptiMEM (Invitrogen) supplemented with GlutaMax (Invitrogen), and 0.01 M HEPES (Invitrogen) and 5% fetal bovine serum (FBS) (CELLect GOLD, MP Biomedical). HT29-Cl.16E cells were grown in Dulbecco’s Modified Eagle Medium (DMEM) (glucose concentration: 4.5g/L) (Invitrogen) supplemented with 10% FBS (Hyclone, Logan, UT) and antibiotics penicillin (100 units/mL) and streptomycin (100 μg/mL) (Invitrogen). Caco-2 cells were grown in MEM with Eagle’s salt and L-glutamine (Invitrogen) supplemented with 20% FBS, non-essential amino acid (NEAA) (Invitrogen), 1 mM sodium pyruvate (Invitrogen), and the antibiotics (Invitrogen). JVM-2 cells were grown in RPMI1640 (Invitrogen) supplemented with 10% FBS (Hyclone, Logan, UT) and the antibiotics (Invitrogen). Vero cells were grown in DMEM supplemented with 5% FBS and the antibiotics (Invitrogen).

For viral culture trials, the cells were rinsed before the inoculation step and maintained with the serum-free medium for each cell line-specific growth medium. Another serum-free medium was DMEM supplemented with antibiotics (Invitrogen), 0.3% tryptose phosphate broth (TPB, Sigma), and 10μg/mL of trypsin (Life Technologies)]. It was used previously in our laboratory for the isolation of porcine epidemic diarrhea virus (PEDV) and designated as "PEDV medium" [37]. It was also used for HuNoV culture trials when HT29-Cl.16E and Caco-2 cells were cultured long-term (~up to 2 months).

IPEC-J2 cells were grown in DMEM/F12 1:1 (Invitrogen) supplemented with 5% FBS and 1% insulin-transferrin-sodium selenite [5μg/mL of bovine insulin, 5 μg/mL of human transferrin and 5 ng/mL of selenium (sodium selenite)] (Sigma), and 5 ng/mL of recombinant human epidermal growth factor (EGF, Invitrogen), and the antibiotics. The cells were rinsed and maintained with DMEM/F12 1:1 (Invitrogen) supplemented with the antibiotics after inoculation with HuNoVs.

Calf small intestinal tissues (jejunum and ileum) were maintained in Cell Crown system (Scaffdex, Finland), and bovine primary cells from ileum mucosal layer were maintained with DMEM supplemented with 10% FBS, GlutaMax, 20 mM HEPES, the insulin-transferrin-sodium selenite, and 10 ng/mL of recombinant human EGF, and the antibiotics. DMEM containing 20% FBS, NEAA, sodium pyruvate, and the antibiotics was also used to maintain calf tissues (duodenum and jejunum) in a petri dish. Bovine PBMCs were grown in E-RPMI (RPMI-1640 supplemented with 8% FBS, 2 mM L-glutamine, 0.1mM NEAA, 1mM sodium pyruvate, 20mM HEPES, 10 μg/mL ampicillin and 100 μg/mL Gentamicin).

### Other supplements

Glycochenodeoxycholic acid (GCDCA) (Sigma, St. Louis, MO, USA) was used in viral culture trials. Bacterial culture supernatants (LB media for *Escherichia coli* Nissle 1917 [Nissle], MRS growth media for *Lactobacillus rhamnosus* GG (ATCC 53103) [LGG], *Lactobacillus reuteri* strain (ATCC 23272) [LR], and *L*. *acidophilus* NCFM™ (ATCC 700396) [LA]), and the supernatants of cultures where these bacteria were incubated with immune cells (i.e., porcine spleen mononuclear cells [MNC]) (Cell+LGG, Cell+Nissle, Cell+LR, and Cell+LA) [38] were also tested as supplements for HuNoV culture trials. They were filtered through 0.2μm-pore size filters, aliquoted and stored at -80°C until use.

Each of the five cell lines, HIEC, HT29-Cl.16E, Caco-2, IPEC-J2, and Vero, were incubated for 2 days with serial 10-fold dilutions of bacteria culture supernatants and the culture supernatants of co-culturing bacteria and MNCs to determine whether there was acute cell toxicity caused by the supernatants (no cell death or significant change in cell morphology indicative of no cell toxicity). From microscopic analysis, an optimal concentration (highest non-toxic concentration) was determined qualitatively.

### Virus propagation conditions

For HuNoV and HuSaV culture trials, confluent HIEC, HT29-Cl.16E, Caco-2, IPEC-J2, and Vero cell monolayers in 24-well plates, or 0.8 x 10^6^ JVM-2 cells/well seeded in six-well plates were used for HuNoV or HuSaV inoculation. Before inoculation, cells were washed with cell specific serum-free medium twice. Then 100μL of inoculum (approx. 10 ^5~8^ copies of viral RNA) per well were added in the 1mL (for 24-well plates) or 3mL (for 6-well plates) serum free media that were optimal for each cell line, or the medium containing the bacteria culture supernatants, or the culture supernatants of co-culturing bacteria and immune cells. 100μL of the culture supernatants from each well were collected at 0 hour post-inoculation (hpi) and stored at -80°C. These cultures were incubated for 4–9 days and then frozen and thawed for three cycles. HuNoV and HuSaV RNA levels in these cell lysates were quantitated by RT-qPCR. The inoculum was not removed after inoculation for these HuNoV and HuSaV culture trials.

For BoNoV culture trials, the organ segments from duodenum, jejunum and ileum were collected aseptically, dissected open longitudinally and washed briefly with serum-free medium to remove intestinal contents and then placed in a petri dish with the mucosal side facing upwards (duodenum and jejunum), and using a Cell Crown^TM^ plate insert (Scaffdex, Finland) in 12-well plates (jejunum and ileum). We chose the Cell Crown system to maintain the mucosal side of a cut tissue in the petri dish. We could not check cell viability, so we only maintained them for a short time (~ 1 day) for BoNoV propagation trials. For the Cell Crown system, 1 mL serum-free medium with or without GCDCA was added to both the inside and outside of the membrane holding the intestinal tissues, and immediately inoculated with 100 μl of GIII.2 BoNoV filtrates (approx. 10^7^ copies of BoNoV RNA). The culture medium from the mucosal side was collected at 0 hpi and after 24 hpi. Primary immune cells (PBMC and intestinal MNC cells) were seeded into 24-well plates (approx. ~10^5^ PBMC cells/well and approx. ~10^6^ MNC cells/well) with 1 mL medium with or without chemicals and immediately inoculated with 100 μL GIII.2 BoNoV filtrate (approx. 10^6^ or 10^7^ copies). These cultures were incubated for 3 days and then frozen and thawed for three cycles. Primary intestinal epithelial cells were seeded into T25 flasks. After reaching confluency (approx. 2 weeks), the growth medium was replaced by 3 mL of serum-free medium with or without chemicals and 100 μL of fecal filtrates containing GIII.2 BoNoV (approx. 10^7^ copies) was added. These cultures were incubated for 5 days and then frozen and thawed for three cycles. For all these BoNoV culture trials, the inocula were not removed after inoculation.

## Results

### HuNoVs and a HuSaV did not grow in various primary and cell lines

We tested six cell lines (HIEC, HT29-Cl.16E, Caco-2, JVM-2, IPEC-J2, and Vero cells) and four HuNoV strains (one GII.2, two GII.4, and one GII.6). GI.1 HuNoV strain was tested in JVM-2 cells. Similarly, six cell lines and one HuSaV strain (GI.2) were tested. HIEC, HT29-Cl.16E, Caco-2 and IPEC-J2 cell lines were selected because of their gastrointestinal origin, although IPEC-J2 is pig origin cells. JVM-2 was also included as a representative human B cell line. Furthermore, a monkey kidney cell line, Vero, was also included because of their versatile capacity to propagate a variety of viruses. As summarized in Table 1, the variation in cell lines had no major impacts on the increase of HuNoV and HuSaV RNA levels as determined by RT-qPCR. Based on these results, we concluded that there was no substantial HuNoV and HuSaV replication under these conditions using the various cells and virus strains.

**Table 1:** Fold changes in HuNoV RNA levels at 96 hpi relative to 0 hpi in cells (cell lysates) inoculated with HuNoV or HuSaV.

Cell name	Species	Cell type	Origin	Inoculated strain	Fold changes
HIEC	Human	Primary cell (epithelia)	Jejunum and Ileum	NoV GII.2 (HS255)	1.2
				NoV GII.4 (HS194)	1.0
				NoV GII.4 (HS288)	1.1
				NoV GII.6 (HS245)	1.4
				SaV GI.2	1.0
HT29-Cl.16E	Human	Continuous cell line (goblet)	Colon	NoV GII.2 (HS255)	0.9
				NoV GII.4 (HS194)	1.0
				NoV GII.4 (HS288)	0.8
				NoV GII.6 (HS245)	0.9
				SaV GI.2	1.2
Caco-2	Human	Continuous cell line (epithelia)	Colon	NoV GII.2 (HS255)	0.9
				NoV GII.4 (HS194)	1.0
				NoV GII.4 (HS288)	1.1
				NoV GII.6 (HS245)	0.9
				SaV GI.2	0.4
JVM-2	Human	Continuous cell line	B-cell	NoV GI.1 (Norwalk)	1.5
				NoV GII.2 (HS255)	0.9
				NoV GII.4 (HS194)	0.4
				NoV GII.4 (HS288)	1.0
				NoV GII.6 (HS245)	0.9
				SaV GI.2	NT
IPEC-J2	Pig	Continuous cell line (epithelia)	Jejunum	NoV GII.2 (HS255)	1.4
				NoV GII.4 (HS194)	0.8
				NoV GII.4 (HS288)	2.0
				NoV GII.6 (HS245)	0.4
				SaV GI.2	0.7
Vero	Monkey	Continuous cell line	Kidney	NoV GII.2 (HS255)	1.2
				NoV GII.4 (HS194)	1.1
				NoV GII.4 (HS288)	0.9
				NoV GII.6 (HS245)	NT
				SaV GI.2	1.1

NT: not tested.

### HuNoV and HuSaV did not grow in the cultures supplemented with bacterial culture supernatants or bacteria-treated immune cell culture supernatants

We also added the bacteria culture supernatants or bacteria-treated MNC to explore whether these bacteria-derived or bacteria-induced MNC factors supported HuNoV and HuSaV growth in cultured cells.

As summarized in Table 2, all tested bacteria supernatants or bacteria-treated MNC culture supernatants had no substantial impact on the viral RNA levels as determined by RT-qPCR except for IPEC-J2 cells. The IPEC-J2 cells treated with bacteria supernatants showed a drastic decrease in HuNoV RNA levels (< 0.1 fold) for unknown reasons. Based on these results, we concluded that there was no obvious HuNoV and HuSaV replication under these test conditions.

**Table 2:** Fold changes in HuNoV and HuSaV RNA levels at 120 hpi relative to 0 hpi in cells (cell lysates) inoculated with HuNoV or HuSaV in medium supplemented with bacteria or the culture supernatants of co-culturing bacteria and immune cells.

Cell name	Inoculated viruses and genotype	Bacteria culture sup				Culture Sup of co-culturing bacteria and immune cells			
		LGG	Nissle	LR	LA	LGG	Nissle	LR	LA
HIEC	NoV GII.4 mix^a^	2.4	1.9	2.0	2.0	1.9	1.9	1.9	1.8
	SaV GI.2	1.5	1.5	1.3	1.8	1.5	1.3	1.7	1.4
HT29-Cl.16E	NoV GII.4 mix^a^	1.7	2.0	2.1	2.2	1.9	1.8	1.7	1.2
	SaV GI.2	1.6	1.5	1.3	1.4	1.4	1.4	1.5	1.5
Caco-2	NoV GII.4 mix^a^	1.8	2.0	1.9	1.7	2.0	2.0	1.9	2.0
	SaV GI.2	2.1	1.5	1.4	1.7	1.7	1.7	1.9	2.0
IPEC-J2	NoV GII.4 mix^a^	<0.1	<0.1	NT	NT	1.2	1.2	2.3	2.1
	SaV GI.2	1.4	1.9	1.2	1.8	1.8	1.7	1.5	1.8
Vero	NoV GII.4 mix^a^	2.0	2.1	2.4	2.0	2.1	1.9	2.0	1.4
	SaV GI.2	1.4	1.3	1.3	1.7	1.7	1.6	0.7	0.4

NT: not tested.

^a^ The mixture of HS66, HS194, HS292, and HS288.

### HuNoVs and a HuSaV did not grow in cell lines transfected with a human small intestinal cDNA library

Semi-confluent Caco-2 and Vero cells grown in four 24-well plates (total 96 wells) were infected with a genetically modified amphotropic retrovirus containing a human small intestine cDNA library and the G418 resistance gene (Clontech-Takara Custom Service) at multiplicity of infection of 1.0 for Caco-2 cells, and 0.1 for Vero cells, respectively. Both cell lines were scaled up in medium containing FBS and G418 (500 μg/mL): one well from a 24-well plate to one well in a 6-well plate, then to a T25 flask. Each T25 flask of Vero (45 flasks in total) and each well of the 6 well plates for Caco2 (82 wells in total) that survived under G418 selection were transferred to a 24-well plate with FBS- and G418-free medium optimal for each cell line, and washed again with fresh FBS- and G418-free medium on the day prior to inoculation with 100 μL of the mixture of GII.4 HuNoVs and a GI.2 HuSaV. The cells were incubated for ~6 days and then underwent three freeze-thaw cycles. RT-qPCR results of both the Caco-2 and Vero cell culture lysates following human GII.4 NoVs or GI.2 SaV infection showed no substantial changes in viral RNA levels compared with the inoculation level (less than 2-fold). Based on these results, we concluded that there was no substantial HuNoV and HuSaV replication under these test conditions. However, the diversity and expression level of the insert sequence from the library in the cells after selection were unknown.

### HuNoV RNA levels were increased in the long-term cultured Caco-2 cells, but the effects were inconsistent

Caco-2 and HT29-Cl.16E cells were cultured in 24-well plates for a prolonged period (until 2 months) with growth medium changed every 3~4 days.

The following condition was tested first: 1 month-cultured Caco-2 and HT29-Cl.16E cells, respectively, were rinsed and 1 mL of FBS-free medium or the PEDV medium [37] was added; the cells were inoculated with the mixture of GII.4 HuNoV positive stool filtrates (100 μL containing approx. 10^5^ copies of HuNoV RNA per well). These cultures were incubated for 6 days, frozen and thawed for three cycles, and then HuNoV RNA levels were quantitated by RT-qPCR. Under these conditions, we observed a 36-fold NoV RNA level increase in the Caco-2 cells cultured with the PEDV medium, whereas the viral RNA level was not increased greatly (1.6-fold) in the FBS-free medium used for other experiments (Table 3). HuNoV RNA levels in HT29.Cl-E1 were similar (2.2-fold) between these two media, and did not increase substantially. We tested further serial passages using the first passage of cell supernatants (approx. 10 ^5^ RNA copies/mL) on 1-month and 2-month cultured Caco-2 cells using the PEDV medium. We also tested the effect of supplementation of GCDCA in this medium.

**Table 3:** Fold changes in HuNoV RNA levels at 6 or 9 dpi relative to 0 hpi in cells (cell lysates) inoculated with HuNoV GII.4 mixture in long-term cultured HT29-Cl.16E and Caco-2 cells.

Cell name	Passage	Cell culture period before inoculation	Medium	Incubation period	No treatment	GCDCA		
				Days post-inoculation (dpi)		50 μM	100 μM	200μM
HT29-Cl.16E	1	1 month	serum free medium	6 dpi	2.2	NT	NT	NT
			PEDV medium	6 dpi	2.2	NT	NT	NT
Caco-2	1	1 month	serum free medium	6 dpi	1.6	NT	NT	NT
			PEDV medium	6 dpi	36	NT	NT	NT
	2	1 month	PEDV medium	9 dpi	28	12	42	69
		2 months	PEDV medium	9 dpi	424	607	238	209

NT: not tested.

The NoV RNA level increase (28-fold increase) in the 1-month cultured Caco-2 cells at 9 dpi was similar to that (36-fold increase) of the primary inoculation on the 1-month cultured Caco-2 cells, whereas that in the 2-month cultured Caco-2 cells showed a 424-fold increase at 9 dpi. For the 2-month cultured Caco-2 cells, the NoV RNA level was slightly higher (607-fold increase) when supplemented with 50 μM GCDCA compared with no GCDCA (424-fold increase) or 100 μM (238-fold increase) or 200 μM GCDCA (209-fold increase). However, this trend was different in the 1-month cultured Caco-2 cells (Table 3). These results indicated that the 2-month cultured Caco-2 cells were more susceptible to the NoV strains tested. Effects of GCDCA on the NoV strains tested suggested that GCDCA may promote virus replication. However, two subsequent trials using another batch of 2-month cultured Caco-2 cells with the same original stools and the CDC HuNoV samples failed to reproduce substantial increase in HuNoV RNA levels.

### BoNoV did not grow in intestinal tissues, primary intestinal cells, intestine- and blood-derived immune cells

Calf intestinal tissues (duodenum, jejunum, and ileum tissue sections) or primary intestinal cells (from ileal mucosa) and immune cells (intestinal MNCs from ileum and PBMC) were inoculated with filtered suspensions of the large intestinal contents of a Gn calf experimentally inoculated with GIII.2 BoNoV. As summarized in Table 4, the variation in tissue/cells had no substantial impacts on the BoNoV RNA levels (≤ 2.6-fold increases) as determined by RT-qPCR. Based on these results, we concluded that there was no substantial BoNoV replication under these conditions.

**Table 4:** Fold changes in BoNoV RNA levels in cells (cell lysates) inoculated with GIII.2/BoNoV/CV-186-OH strain in various calf primary cells and tissues.

Cell type	Origin	Incubation period	Fold Change
Primary Epithelial Cells	Ileum	120 h	1.1
MNC	Ileum	72 h	1.5
PBMC	Blood	72 h	1.2
Tissue (in Petri dish)	Duodenum	24 h	0.8
	Jejunum	24 h	0.5
Tissue (in Cell Crown system)	Jejunum	24 h	1.2
	Ileum	24 h	2.6

NT: not tested.

## Discussion

In this study, we attempted to grow HuNoVs, a HuSaV, and a BoNoV in cultured continuous cell lines, primary cells, or intestinal tissues. For HuNoVs and the HuSaV, we tested various cell lines (HIEC, HT29-Cl.16E, Caco-2, IPEC-J2, JVM-2 and Vero cells), and NoV strains (GI.1, GII.2, GII.4, and GII.6).

HIEC, HT29-Cl.16E and Caco-2 are human origin cell lines. They were chosen because of their gastrointestinal origin. We also included a human B cell line JVM-2 based on a recent publication reporting that B cells in the lamina propria of duodenum stained positive for HuNoV antigens in a chimpanzee challenge model [39]. During our study Jones et al. reported the propagation of a HuNoV strain in human B cells, BJAB and Raji [11, 17]. They showed that bacteria enhanced HuNoV replication in B cells using a non-filtered stool suspension (i.e., presence of enteric bacteria) or adding synthetic HBGA or HBGA-expressing bacteria to filtered bacteria-free inocula. It will be interesting to test these conditions to examine the ability of HuNoV to propagate in JVM-2 cells in future studies. We also used a porcine small intestinal epithelial cell line, IPEC-J2, because of their gastrointestinal origin and our experimental data showing that Gn pigs are susceptible to GII.4 HuNoV infection [40]. Furthermore, Vero, a monkey kidney cell line derived from a non-human primate species, was also included because of their versatile capacity to propagate a variety of viruses including caliciviruses [41, 42] and their interferon pathway deficiency [43]. Recent studies demonstrated that the interferon pathway affects HuNoV replication *in vitro* and *in vivo* [44, 45]; however, a limited role of the epithelial IFN responses in host control of HuNoV RNA replication has also been reported [46].

We also did not detect increased HuNoV RNA levels in JVM-2, a B cell line. Furthermore, no HuNoV and HuSaV RNA titer changes were detected in human small intestine cDNA-transfected Vero and Caco-2 cells. Although we selected long insert cDNA (≥(1.0 kb) for cloning into the retrovirus, the sequences of cDNA inserts and their expression as well as their diversity in the cells after selection were unknown.

Another condition tested only for HuNoVs that resulted in a substantial increased HuNoV RNA level was the long-term (1 and 2 months) culturing of Caco-2 cells using the PEDV medium [37]. This FBS-free medium differs from that used in our original experiments. The reason why we tested additional conditions were that: 1) we obtained another HuNoV GII.4 positive stool specimen; 2) we maintained Caco-2 and HT29-Cl.16E cells in 24-well plates for a prolonged period; and 3) we isolated PEDV using the PEDV medium (DMEM supplemented with TPB and trypsin) that was not used in our previous HuNoV culture trials [37].

HuNoV RNA levels were Increased 36-fold in the first inoculation trial of 1 month cultured Caco-2 cells and this increased level was the highest among our studies at that stage. Next, we tried secondary passage by inoculating the first passage (P1) sample onto other 1- and 2-month cultured Caco-2 cells. HuNoV RNA levels increased 28-fold in the 1-month cultured Caco-2 cells. They increased to higher levels when the culture medium was supplemented with 100 μM (42-fold) or 200 μM GCDCA (69-fold). HuNoV RNA levels were another log higher (424-fold) when the first passage was performed in the 2-month cultured Caco-2 cells, and increased further (606-fold) when the culture was supplemented with 50 μM GCDCA (Table 3), showing that GCDCA may have enhancement effect on HuNoV replication in Caco-2 cells; however, this positive result was inconsistent. This was consistent with the requirement of bile acids for the growth of porcine SaV Cowden strain in cell culture [22], and certain strains of HuNoVs in human enteroids [18]. We did not verify our results by other methods (e.g. nonstructural protein expression, dsRNA detection, (-) strand RNA detection, etc). Unfortunately, we could not reproduce the HuNoV RNA increases in the 2^nd^ and 3^rd^ trials using different batches of long-term cultured Caco-2 cells, the same HuNoV mixture as the inocula as in Table 3, and additional 27 individual stool suspensions provided from CDC. The replication of HuNoV in 3D cultured Caco-2 cells and a clone of Caco-2 cells, C2BBe1, has been reported [15, 16]. The increased NoV RNA levels detected in this study (1.0–2.8 Log_10_ increases) were similar to those in the previous reports [15, 16]. Caco-2 cells have been widely used for HuNoV culture trials and most research groups including our group could not grow HuNoV previously [31, 47–49]. This inconsistency may be explained by the differences in stool suspension batch, HuNoV strains, medium lots and cell culture conditions, because we did not observe increased NoV RNA levels in the 2^nd^ and 3^rd^ trials. Further experiments are required to explore the factor(s) essential for HuNoV replication. During the preparation of our manuscript, another new HuNoV culture system using human intestinal enteroids was reported [18]. The increased HuNoV RNA levels in the Caco-2 cells and their derivative cells, B cells, and human intestine enteroids were similar (ranging from 2–3 log_10_ levels) based on qRT-PCR. We did not test HuNoV isolates known to grow in either the BJAB cells or enteroids culture systems, but used the same genogroups / genotypes of HuNoVs in this study.

BoNoV did not propagate in any test conditions including using bovine intestinal tissues where BoNoV replicates *in vivo* [28]. The failure of BoNoV to replicate in intestinal tissues may be due to the difficulty in maintaining the Gn calf-derived tissues healthy in *ex vivo* condition for a prolonged time under our protocol. The medium in the petri dish or wells became cloudy at several hours post BoNoV inoculation. This was likely due to the degradation of tissue. FBS in the medium used under several conditions including with MNCs and PBMCs, may also interfere with virus infection/replication. We could not detect any substantial increase of BoNoV RNA levels in primary calf cells from ileal mucosa under the test conditions. The cell type(s) in which GIII.2 BoNoVs replicate *in vivo* is still unknown [28], although GIII.1 BoNoV Jena strain likely replicates in enterocytes, because the capsid protein was detected in BoNoV infected villi [50]. Establishment of BoNoV culture systems *in vitro* needs to be investigated in future studies.

Establishment of an efficient NoV and SaV cultivation system is critical to elucidate virus stability and inactivation conditions, viral infection/replication mechanisms including receptor identification, and to develop antiviral drugs as well as attenuated vaccines. Efficient and inexpensive cell culture systems for human and animal NoVs and SaVs are still lacking, except for murine NoVs and porcine SaV Cowden strain.

HuNoV replication levels observed in this study and by other groups [11, 15–18, 51] are still low compared to those achieved for murine NoVs [12] and porcine SaVs [52]. Current culture systems (BJAB cells or primary human stem cell-derived enteroids) for HuNoVs support modest virus replication. Some of the approaches used in our study may be used to investigate if they can promote HuNoVs replication in the above two systems. Further trials under different culture conditions, as well as using other untested cell lines from other tissues, introducing specific cDNA inserts, induction/suppression of specific pathway(s), or extensive serial passages as used for the isolation of porcine SaV Cowden strain [24, 25] may aid in the isolation of HuNoV and HuSaV as well as other animal NoVs and SaVs.
